# The ‘Practical Approach to Lung Health’ in sub-Saharan Africa: a systematic review

**DOI:** 10.5588/ijtld.15.0613

**Published:** 2016-04-01

**Authors:** H. Banda, R. Robinson, R. Thomson, S. B. Squire, K. Mortimer

**Affiliations:** * Research for Equity and Community Health Trust, Lilongwe, Malawi; † Department of Clinical Sciences and Centre for Applied Health Research and Delivery, Liverpool School of Tropical Medicine, Liverpool, UK

**Keywords:** PALSA, PALM/LHW, training, tuberculosis, non-communicable disease

## Abstract

SETTING: There is a high burden of respiratory disease in sub-Saharan Africa. To address this problem, the World Health Organization launched the ‘Practical approach to Lung Health’ (PAL), i.e., locally applicable integrated syndromic algorithms, to improve primary care management of these diseases.

OBJECTIVE: To examine the evidence for the impact of PAL on the diagnosis and management of tuberculosis (TB) and other common respiratory problems in sub-Saharan Africa.

DESIGN: A systematic review of MEDLINE (1998–2015), EMBASE (1998–2015) and CINAHL (1998–2015) was conducted to find trials evaluating PAL implementation in sub-Saharan Africa.

RESULTS: Five studies were found, evaluating three PAL variations: PAL in South Africa (PALSA), PALSA with integrated human immunodeficiency virus treatment (PALSA PLUS) and PAL in Malawi using lay health workers (PALM/LHW). PALSA increased TB diagnosis (OR 1.72, 95%CI 1.04–2.85), as did PALSA PLUS (OR 1.25, 95%CI 1.01–1.55). Cure or completion rates in retreatment cases in PALSA and PALSA PLUS were significantly improved (OR 1.78, 95%CI 1.13–2.76). PALM/LHW, which examined TB treatment success, found no significant improvement (*P* = 0.578).

CONCLUSION: The limited research performed shows that PAL can be effective in TB diagnosis and partial treatment success; however, more evidence is needed to assess its effects on other respiratory diseases, especially in wider sub-Saharan Africa.

## RESUME

CONTEXTE : L'Afrique subsaharienne est durement touchée par les affections respiratoires. C'est en réponse à ce problème que l'Organisation Mondiale de la Santé a lancé l' « Approche pratique de la santé respiratoire » (PAL) ; ce sont des algorithmes syndromiques intégrés et applicables localement afin d'améliorer la prise en charge initiale de ces maladies.

OBJECTIF : Examiner les preuves de l'impact du PAL sur le diagnostic et la prise en charge de la tuberculose (TB) et d'autres problèmes respiratoires fréquents en Afrique sub-saharienne.

SCHÉMA : Une revue systématique de MEDLINE (1998–2015), d'EMBASE (1998–2015) et de CINAHL (1998–2015) a été réalisée à la recherche d'essais évaluant la mise en œuvre du PAL en Afrique sub-saharienne.

RÉSULTATS : Cinq études ont été trouvées évaluant trois variantes du PAL : PAL en Afrique du Sud (PALSA), PALSA avec intégration du traitement du virus de l'immunodéficience humaine (PALSA PLUS) et PAL au Malawi grâce à des prestataires de soins des communautés (PALM/LHW). PALSA a accru le diagnostic de TB (OR 1,72 ; IC95% 1,04–2,85), comme l'a fait PALSA PLUS (OR 1,25 ; IC95% 1,01–1,55). Le taux de guérison ou d'achèvement du traitement des cas de retraitement a également été significativement amélioré grâce à PALSA et PALSA PLUS (OR 1,78 ; IC95% 1,13–2,76). PALM/LHW, qui a examiné le taux de succès du traitement de la TB, n'a pas mis en évidence d'amélioration significative (*P* = 0,578).

CONCLUSION : La recherche limitée qui a été réalisée montre que le PAL peutêtre efficace en matière de diagnostic de la TB et partiellement en matière de succès du traitement ; il faut cependant davantage de preuves pour son impact sur les autres affections respiratoires, surtout dans d'autres pays d'Afrique sub-saharienne.

## RESUMEN

MARCO DE REFERENCIA: EnÁfrica subsahariana existe una alta carga de morbilidad por enfermedades respiratorias. Con el propósito de responder a esta situación la Organización Mundial de la Salud puso en marcha la estrategia ‘Enfoque práctico de la salud pulmonar’ (PAL); el enfoque consiste en una serie de algoritmos sindrómicos integrados, encaminados a mejorar la coordinación asistencial de la atención primaria de las enfermedades respiratorias.

OBJETIVO: Examinar los resultados de la estrategia PAL en el diagnóstico y el tratamiento de la tuberculosis (TB) y otros problemas respiratorios frecuentes en África subsahariana.

MÉTODOS: Se llevó a cabo una revisión sistemática de la bibliografía en las bases de datos MEDLINE (1998–2015), EMBASE (1998–2015) y CINAHL (1998–2015) en busca de estudios sobre la aplicación de la estrategia PAL en África subsahariana.

RESULTADOS: Se encontraron cinco estudios que evaluaban tres variaciones de la estrategia PAL, a saber, PAL en Suráfrica (PALSA), PALSA con tratamiento integrado de la infección por el virus de la inmunodeficiencia humana (PALSA PLUS) y PAL en Malawi aplicado por trabajadores de salud no especializados (PALM). La aplicación de PALSA aumentó el diagnóstico de la TB (OR 1,72; IC95% 1,04–2,85), de igual manera que la ejecución de PALSA PLUS (OR 1,25; IC95% 1,01–1,55). Con PALSA y PALSA PLUS se mejoró de manera significativa la tasa de curación y de compleción del tratamiento en los casos de retratamiento (OR 1,78; IC95% 1,13–2,76). El estudio sobre PALM, que examinó eléxito terapéutico, no encontró un progreso significativo (*P* = 0,578).

CONCLUSIÓN: La escasa investigación realizada puso en evidencia que la estrategia PAL puede ser eficaz en el diagnóstico de la TB y ofrecer unéxito parcial en materia de tratamiento, pero se precisa más información sobre sus efectos en otras enfermedades respiratorias y sobre todo una visión más amplia en África subsahariana.

LUNG DISEASE is a leading cause of mortality in low- to middle-income countries, where it is reported to account for 15% of all deaths.^1^ A large proportion of these are attributable to tuberculosis (TB). The World Health Organization (WHO) estimates that there were 9 million TB cases in 2013, a quarter of which were in Africa, where numbers of cases and rates of death relative to the population are highest.^2^ Alongside TB, non-communicable respiratory conditions are increasingly recognised as major health problems in these settings.^3^ Sub-Saharan Africa has an estimated 50 million people with asthma^4^ and an unknown prevalence of chronic obstructive pulmonary disease (COPD). In 1990, COPD was thought to affect 4.41 per 1000 men and 2.49/1000 women.^5^ The WHO predicts that as life expectancy rises in Africa, deaths due to COPD and other non-communicable diseases will increase by 27% by 2030.^6^

In response to a pressing need for wide-ranging action, the WHO launched the ‘Practical Approach to Lung Health’ (PAL) in 1998, updated in 2005, which aims to improve the diagnosis and treatment of common respiratory diseases, including pneumonia, TB, asthma and COPD, in patients aged >5 years.^7^ The approach involves strategies to strengthen and overcome weak health systems through the implementation of locally applicable integrated syndromic algorithms for the detection and management of respiratory disease in primary care. The global aim is to improve case management and coordination among health workers of different levels.^7^

In sub-Saharan African countries, many primary care health care providers are not trained physicians but nevertheless perform many of the diagnostic and clinical functions of qualified doctors.^8^ There are thus shortfalls in skills and knowledge in the recognition, diagnosis and management of respiratory diseases. PAL aims to empower primary care practitioners to make diagnoses, perform initial investigations, identify patients requiring emergency care or immediate referral and commence appropriate initial treatment.^9^ Educational support and materials are key components of this approach.

This systematic review set out to collect evidence of the effectiveness of PAL in improving the recognition, diagnosis and management of lung disease in sub-Saharan Africa, with a view to identifying knowledge gaps and areas for further research.

## STUDY POPULATION AND METHODS

A comprehensive literature search of MEDLINE (1998–June 2015), EMBASE (1998–June 2015) and CINAHL (Cumulative Index to Nursing and Allied Health Literature; 1998–June 2015) was performed using a protocol-driven search strategy (full protocol available on request). Search terms for ‘Practical approach to lung health’ included PAL, practical approach to lung health, practical approach to lung health in South Africa, PALSA. These were combined with terms for Africa, sub-Saharan and the name of each of the 47 sub-Saharan African countries.

References from published reviews and included publications were reviewed, as were abstracts from proceedings of the major conferences, including the British Thoracic Society (BTS), the European Respiratory Society (ERS), the American Thoracic Society (ATS), the International Union Against Tuberculosis and Lung Disease (The Union) and any thoracic medicine conferences specific to sub-Saharan Africa within the last 2 years. Clinical trial registers were searched. There were no restrictions on language.

The following inclusion criteria were used: 1) applies the WHO PAL programme, 2) sample population was from sub-Saharan Africa, and 3) outcome related to the diagnosis or management of lung disease.

Studies were initially assessed for inclusion on the basis of their titles and abstracts. Full texts of studies potentially meeting the inclusion criteria were then obtained and screened by two independent reviewers (HB and RR). A third author (KM) reviewed the list and adjudicated discussions about study inclusion in case of disagreement.

Data extraction was performed by two independent reviewers (HB and RR) using a standardised data extraction form. The study design, sample population, intervention, study objectives, statistical analysis and outcomes were then collated. Two independent reviewers (HB and RR) assessed the methodological quality of the studies using the Cochrane ‘risk of bias tool’^10^ for randomised controlled trials and the Newcastle-Ottawa scale for observational studies.^11^ These are evidence-based systematic tools for evaluating the internal validity of studies. A maximum score of six was used for cross-sectional studies and nine for cohort studies. A narrative synthesis was then created from these papers.

## RESULTS

The initial search identified 47 papers published between 1998 and June 2015. Nine papers were excluded as they were not relevant to the aim of the review after the titles and abstracts had been screened for eligibility. A total of 38 full-text articles were then assessed, five of which met the inclusion criteria. The most common reasons for exclusion were not having a sub-Saharan African sample population or outcomes focused on issues encountered during PAL implementation rather than the impact on diagnosis or management of lung disease. The five studies included are summarised in Table 1.^12^–^16^ Although out of the scope of the review, the studies relevant to PAL implementation are also summarised, as they provide additional insights into the use of these tools in sub-Saharan Africa (Table 2, Figure).^9^,^17^–^25^

**Table 1:**
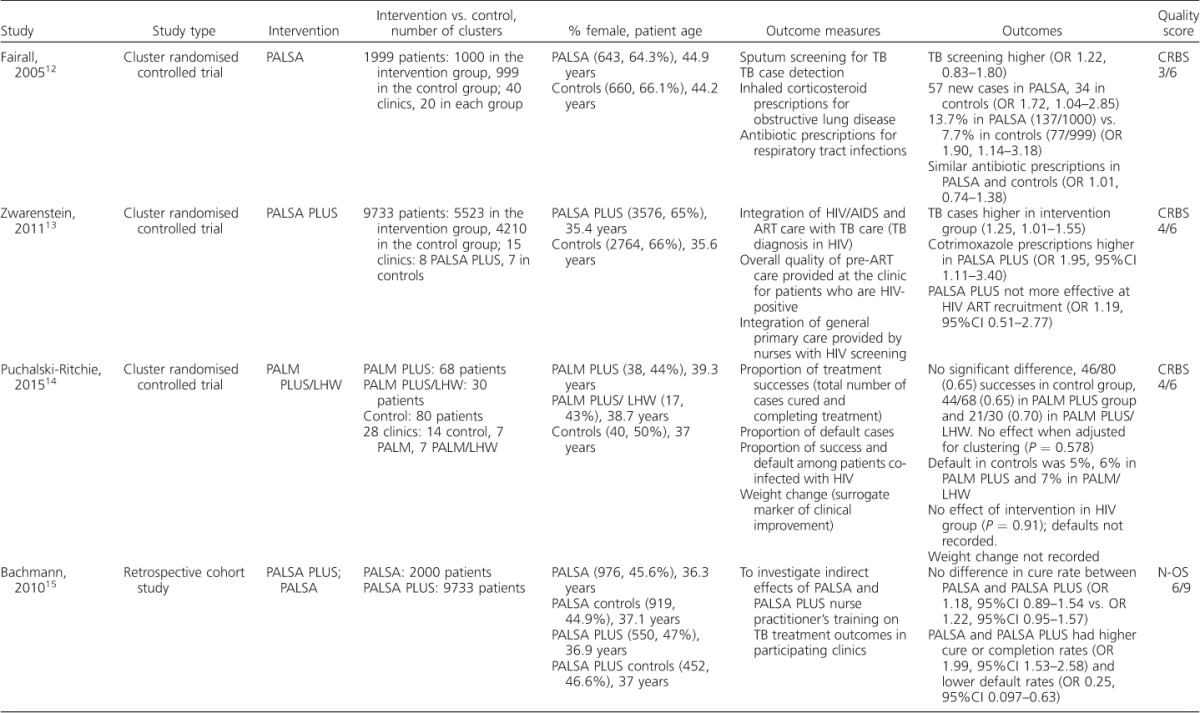
Studies included in the review

**Table 1:**
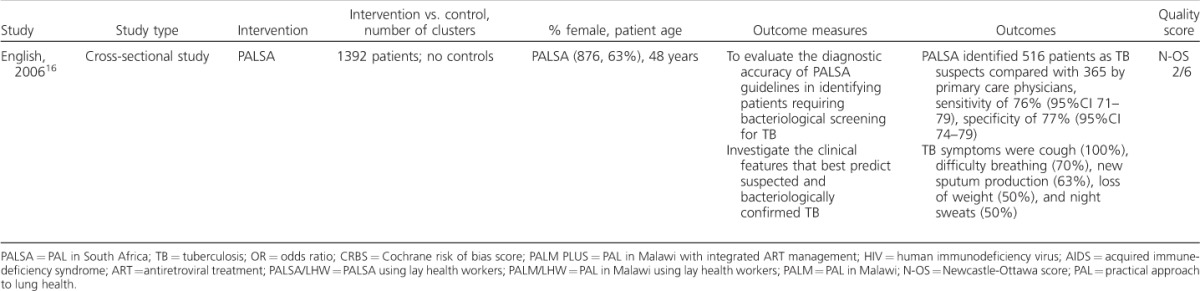
(continued)

**Table 2:**
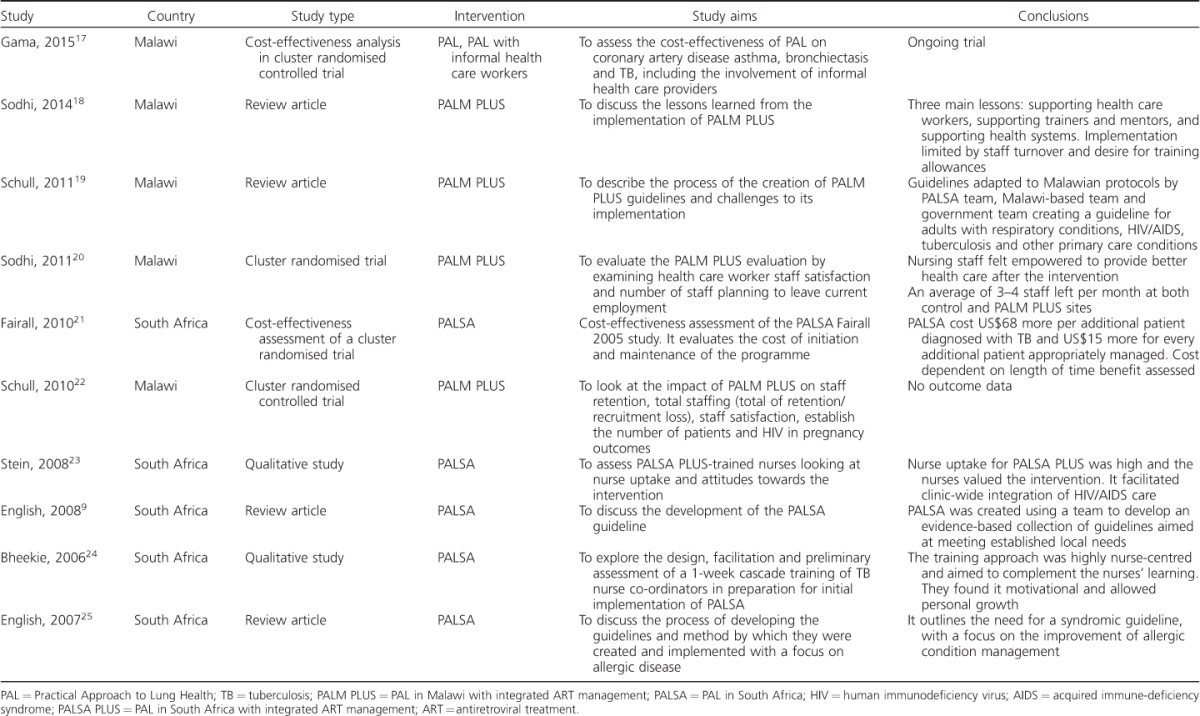
Studies excluded from the review but relevant to PAL implementation in sub-Saharan Africa

**Figure:**
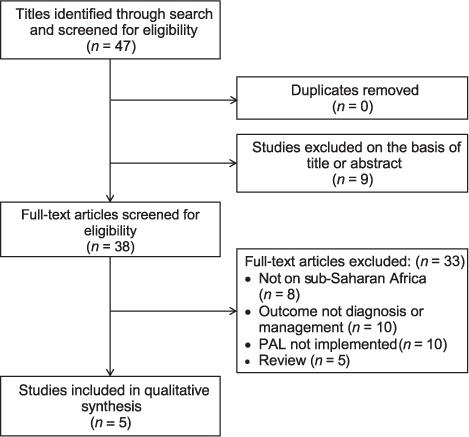
PRISMA flow diagram. PAL = Practical Approach to Lung Health; PRISMA = Preferred Reporting Items for Systematic Reviews and Meta-Analyses.

The five studies included were published between 2005 and 2015 and included three cluster randomised controlled studies, one retrospective cohort study and one cross-sectional study. All aimed to evaluate the impact of PAL on the diagnosis and management of lung disease.

### PAL implementation

Two of the studies used PAL in South Africa (PALSA)^12^ and two implemented PALSA PLUS,^13^ which integrates antiretroviral therapy (ART). One study applied the PAL in Malawi integrating human immunodeficiency virus (HIV) care (PALM PLUS), in which half were allocated to PALM PLUS with ‘lay health workers intervention’ (PALM/LHW).^14^

### Population sample

Four of the study sample populations were from South Africa and one was from Malawi. The two PALSA-controlled trials had similar sized intervention and control groups and a similar sex ratio. The PALM/LHW study had a smaller sample size, and was underpowered as a result.^14^

The PALSA trials had a higher number of women in both the intervention and control groups,^12^,^13^ with the exception of Bachmann et al.^15^ Patients in Fairall et al.,^12^ a PALSA study, were older (average age, intervention group 44.9 years vs. controls 44.2 years) than in Zwarenstein et al.,^13^ the PALSA PLUS trial (35.4 vs. 35.6 years) and PALM/LHW^14^ (PALM 39.3 years, PALM/LHW 38.7 years, controls 37 years).

In the PALSA randomised controlled trials,^12^,^13^ all patients who presented to primary care centres reporting a cough or difficulty breathing in the last 6 months were included (1999 in PALSA^12^ based at 40 clinics, and 9733 in PALSA PLUS at 15 centres).^13^ Cluster randomisation was performed in all controlled studies at the clinic level; PALM/LHW was stratified for ART initiation site.^14^ As PALSA PLUS was based at ART clinics, all patients had HIV.^13^ The PALM/LHW trial was embedded in 14 PALM PLUS control clinics:^22^ 7 used PALM and 7 used PALM/LHW. Outcome data were collected from the national TB register and TB treatment cards. Bachmann et al. retrospectively selected their analysis sample cross-referring patients in PALSA (*n* = 2000) and PALSA PLUS (*n* = 9733) with the national TB register.^15^ The English et al. study was a single-centre cross-sectional study among patients presenting consecutively with cough or difficulty in breathing (*n* = 1392).^16^

### Diagnosis and management of lung disease

All five studies used TB diagnosis or management as a primary outcome. PALSA found sputum sampling (used as a surrogate for case detection) higher in the intervention arm but not significantly so (odds ratio [OR] 1.22, 95%CI 0.83–1.80), but did diagnose significantly more TB cases, 57 compared to 34 in the control clinics (OR 1.72, 95%CI 1.04–2.85).^12^ PALSA PLUS also reported that TB was more likely to be diagnosed among patients attending ART clinics (OR 1.25, 95%CI 1.01–1.55).^13^

The primary outcome of PALM/LHW was TB treatment success. It reported a small, but not statistically significant, increase in treatment success in the intervention group when the data were adjusted for the effects of clustering (*P* = 0.578).^14^ There was no difference in ‘default from treatment’ between the groups.

Bachmann et al. found that the intervention groups were more likely to be smear-positive in both PALSA and PALSA PLUS;^15^ however, this was non-significant. The proportion of patients undergoing retreatment did not differ between the intervention groups in either study (*P* = 0.81 and 0.90, respectively). Overall, neither intervention provided a better outcome; however, the adjusted odds of ‘default from treatment’ was 38% lower in PALSA (a significant result) and 31% lower in PALSA PLUS.^15^ Among retreatment cases, Bachmann et al. reported the odds of cure or completion as 23% higher in PALSA and 78% higher in PALSA PLUS.^15^

The study by English et al. investigated the diagnostic accuracy of staff using PALSA in diagnosing TB.^16^ The nurse was able to identify 37% of patients as TB suspects, whereas the primary care physicians identified 26%, with a sensitivity of 76% (95%CI 71–79) and a specificity of 77% (95%CI 74–79). A nurse using PALSA to detect proven TB was 90% sensitive (95%CI 76–97) and 65% specific (95%CI 63–68). The diagnostic accuracy of the nurse using the guideline in suspected TB compared to the respiratory physicians' diagnosis was 73% sensitive (95%CI 67–78) and had a specificity of 71% (95%CI 68–74).^16^

The PALSA trial also considered other important lung disease management outcomes. A higher proportion of severely unwell patients were referred to a physician (27/257, 10.5% vs. 8/166, 4.8%; OR 2.59, 95%CI 1.06–6.19), and more inhaled corticosteroids were prescribed (137/1000, 13.7% vs. 77/999, 7.7%; OR 1.90, 95%CI 1.14–3.18).^12^

Similar levels of cotrimoxazole were prescribed among HIV patients with TB in both groups in PALSA (13/167, 7.8% vs. 11/147, 7.5%), and rates of antibiotic prescription did not differ (OR 1.01, 95%CI 0.74–1.38).^12^

### Methodological quality

The three randomised control trials were assessed using the Cochrane risk of bias tool (Table 1).^10^ All three had limitations in their approach to intervention allocation concealment. In PALSA, an open random allocation schedule was used, while in PALSA PLUS and PALM/LHW, the concealment is not clearly described. The trials note that blinding was not possible due to the nature of the interventions; however, PALSA reports that it could blind the patients. There is an unexplained loss to follow-up of 70 individuals in the intervention group and 73 in the control group in PALSA.^12^ Several outcomes in PALM/LHW were not reported due to insufficient data being collected.

The Newcastle-Ottawa observational assessment scale^11^ was used to assess the studies by Bachmann et al.^15^ and English et al.^16^ The retrospective study by Bachmann et al.^15^ scored 6/9, as the outcome was available before the study began. English et al. scored 2/6, as there was no control population and the study used a non-random sampling method, leaving potential for bias.^16^

## DISCUSSION

We have found that the PAL strategy provides an opportunity to improve the case detection and management of TB, and there are indications that it could improve general lung health. However, little research has been conducted to evaluate its impact in sub-Saharan Africa. The majority of studies to date have focused on PAL implementation in South Africa, with only one trial taking place outside this country. The two largest studies, PALSA and PALSA PLUS, show that PAL can be effective in increasing TB diagnosis. It also improved the referral of severely unwell patients, and suggested improved management of other lung diseases, as evidenced by the increased prescription of inhaled corticosteroids in PALSA.^12^ In contrast, the PALM/LHW trial found no significant difference from either PAL intervention.^14^

Concerning the PALSA guidelines, English et al. found that PALSA was effective in allowing nurses to identify patients who require bacteriological screening for pulmonary TB with high accuracy when compared with physicians using better investigative techniques.^16^ Bachmann et al. reports no significant difference in TB outcomes in the intervention groups; however, they do report reduced ‘default from therapy’ rates and increased cure and completion rate in retreatment cases.^15^ In general, our findings show that PAL can be adapted to local settings and extended to integrate other diseases to improve health care delivery where implemented.

In 2007, the WHO evaluation of PAL found that 31 countries were at different stages of programme development and implementation.^26^ They reported that the approach was consistent with ongoing health sector development and had enabled the empowerment of health care workers to make appropriate decisions and develop a strong patient-centred approach. There was also a significant reduction in drug prescriptions and the establishment of a method for systematic monitoring of patients. It did, however, note difficulties in defining its relationship with other global respiratory disease initiatives such as the Global Initiative for Asthma (GINA)^27^ and the Global Initiative for Chronic Obstructive Lung Disease (GOLD).^28^ They also reported that advocacy for PAL was lacking and that information on epidemiological impact and cost-effectiveness was limited.^26^

Since the WHO report, several PAL evaluations have been performed globally, as summarised by Hamzaoui & Ottmani,^29^ who established that PAL was effective in reducing the overall number of drugs prescribed per prescription at PAL facilities, and reduced the prescription of antibiotics in most countries, while increasing prescriptions for inhaled corticosteroids. The reduction in antibiotics prescribed is not widespread, and the authors suggest that it is due to pre-guideline prescribing habits. Evidence on the next level of care referrals was mixed.^29^ Since Hamzaoui & Ottmani's structured review, PALM/LHW is the only new trial to assess its clinical impact in sub-Saharan Africa.^14^ This was embedded in the larger PALM PLUS trial, which focused on health care worker retention and job satisfaction.^22^

The present review was structured using protocol-driven quality assessment in line with MOOSE (Meta-Analyses and Systematic Reviews of Observational Studies) guidelines.^30^ The main limitation of this review is the scarcity of available evidence. The majority of the research focused on South Africa, and may not be directly applicable to differing health care systems. South Africa has an established network of nursing staff providing primary care.^12^ Elsewhere in sub-Saharan Africa, this network is chronically understaffed, and the health care worker to population ratio is the lowest globally.^31^ Clinics tend to be run by nurses, clinical officers and medical assistants, many with limited knowledge of lung disease management.^22^ PALM/LHW, focusing on educating lay individuals in understaffed areas, is the only study to address this.^14^ A larger trial, however, is required to establish a definitive answer on its effectiveness.

There is a great need for evaluation of more sub-Saharan African interpretations of PAL. The evidence at the moment is encouraging, but limited. We have found a large evidence gap in the impact of PAL, specifically on respiratory conditions other than TB, such as asthma and COPD. Although PAL can be adapted to suit the local context, the interventions reviewed do not include spirometry as an essential component, despite the fact that it is required to diagnose COPD. Nevertheless, as Hamzaoui & Ottmani demonstrate, PAL has been implemented successfully worldwide. We therefore recommend more research into the barriers to its implementation in sub-Saharan Africa. Studies given in Table 2 provide an insight into how this could be addressed.^29^

## CONCLUSIONS

There is evidence from studies conducted in South Africa that PAL is effective in improving TB diagnosis. There is less evidence relating to other possible impacts, including the management of non-communicable respiratory diseases. One underpowered study has been conducted in the wider sub-Saharan African region. Future research will need to address these substantial gaps in evidence and, in particular, to address the needs of under-served people with chronic respiratory symptoms living in sub-Saharan Africa outside of South Africa.
